# (7a*R*)-1-[(2*R*,5*S*,*E*)-6-Hy­droxy-5,6-dimethyl­hept-3-en-2-yl]-7a-methyl­hexa­hydro-1*H*-inden-4(2*H*)-one

**DOI:** 10.1107/S1600536812051343

**Published:** 2013-01-12

**Authors:** Marcos L. Rivadulla, Massene Sene, María González, Berta Covelo

**Affiliations:** a Dpto. Química Orgánica, Facultade de Química, Universidade de Vigo, E-36310 Vigo, Spain; b Unidad de Difracción de Raios X de Monocristal, Servicio Determinación Estructural, Proteómica e Xenómica, CACTI-Universidade de Vigo, E-36310 Vigo, Spain

## Abstract

The chiral title compound, C_19_H_32_O_2_, contains a [4.3.0]-bicyclic moiety in which the shared C—C bond presents a *trans* configuration and a side chain in which the C=C double bond shows an *E* conformation. The conformations of five- and six-membered rings are envelope (with the bridgehead atom bearing the methyl substituent as the flap) and chair, respectively, with a dihedral angle of 4.08 (17)° between the idealized planes of the rings. In the crystal, the mol­ecules are self-assembled *via* classical O—H⋯O hydrogen bonds, forming chains along [112]; these chains are linked by weak non-classical C—H⋯O hydrogen bonds, giving a two-dimensional supra­molecular structure parallel to (010). The absolute configuration was established according to the configuration of the starting material.

## Related literature

The title compound is a precursor of the hormonally active form of vitamin D3. For general background to vitamin D3, see: Heaney (2008 ▶); Henry (2011 ▶). For related structures, see: Maehr & Uskokovic (2004 ▶). For puckering parameters, see: Cremer & Pople (1975 ▶).
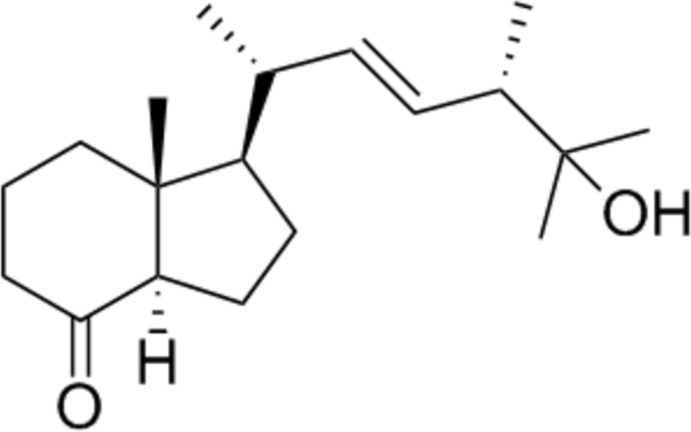


## Experimental

### 

#### Crystal data

C_19_H_32_O_2_
*M* *_r_* = 292.45
Monoclinic, 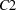

*a* = 20.057 (4) Å
*b* = 7.3816 (15) Å
*c* = 13.700 (3) Å
β = 112.324 (4)°
*V* = 1876.3 (6) Å^3^
*Z* = 4
Mo *K*α radiation
μ = 0.07 mm^−1^
*T* = 293 K
0.45 × 0.36 × 0.18 mm

#### Data collection

Bruker SMART 1000 CCD diffractometer
Absorption correction: multi-scan (*SADABS*; Sheldrick, 1996 ▶) *T*_min_ = 0.602, *T*_max_ = 0.745
4958 measured reflections
3254 independent reflections
2389 reflections with *I* > 2σ(*I*)
*R*_int_ = 0.018

#### Refinement

*R*[*F* ^2^ > 2σ(*F* ^2^)] = 0.048
*wR*(*F* ^2^) = 0.152
*S* = 1.02
3254 reflections
196 parameters
1 restraint
H-atom parameters constrained
Δρ_max_ = 0.14 e Å^−3^
Δρ_min_ = −0.11 e Å^−3^

### 

Data collection: *SMART* (Bruker, 1998 ▶); cell refinement: *SAINT* (Bruker, 1998 ▶); data reduction: *SAINT*; program(s) used to solve structure: *SHELXS97* (Sheldrick, 2008 ▶); program(s) used to refine structure: *SHELXL97* (Sheldrick, 2008 ▶); molecular graphics: *PLATON* (Spek, 2009 ▶) and *Mercury* (Macrae *et al.*, 2006 ▶); software used to prepare material for publication: *SHELXTL* (Sheldrick, 2008 ▶).

## Supplementary Material

Crystal structure: contains datablock(s) I, global. DOI: 10.1107/S1600536812051343/pv2614sup1.cif

Structure factors: contains datablock(s) I. DOI: 10.1107/S1600536812051343/pv2614Isup2.hkl

Supplementary material file. DOI: 10.1107/S1600536812051343/pv2614Isup3.cml

Additional supplementary materials:  crystallographic information; 3D view; checkCIF report

## Figures and Tables

**Table 1:** Hydrogen-bond geometry (Å, °)

*D*—H⋯*A*	*D*—H	H⋯*A*	*D*⋯*A*	*D*—H⋯*A*
O7′—H7′⋯O4^i^	0.82	2.08	2.876 (3)	164
C3*A*—H3*A*1⋯O7′^ii^	0.98	2.56	3.523 (3)	166

Symmetry codes: (i) 
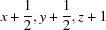
; (ii) 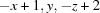.

**Fig. 1.**
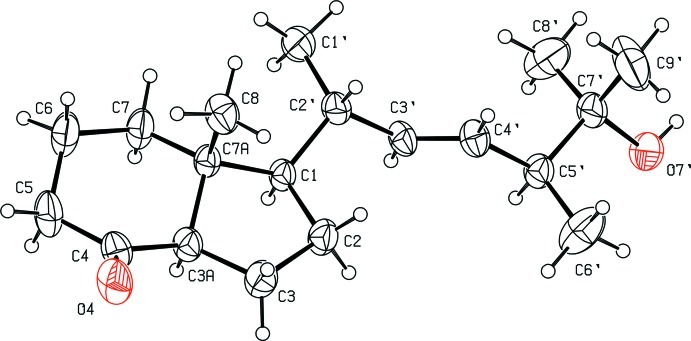
The molecular structure of the title compound. Non-H atoms are present as displacement ellipsoids at the 30% probability level.

**Fig. 2.**
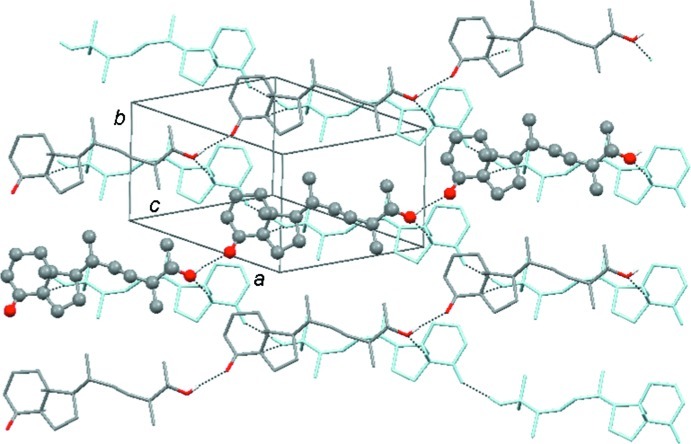
View of two-dimensional supramolecular organization in the crystal structure of the title compound. H atoms not involved in hydrogen bonds (dashed lines) have been omitted for clarify.

## supplementary crystallographic information

### Comment

The title compound is a precursor of 1α,25-dihydroxyvitamin D3 (calcitriol) analogue which is the hormonally active form of vitamin D3. Besides regulating calcium homeostasis, this form is also involved in other cellular processes such as cell differentiation; immune system regulation and gene transcription (Henry, 2011). Nevertheless, the clinical utility of this hormone for treatment of cancers and skin disorders is limited by its hypercalcemic effects (Heaney, 2008), for this purpose the design and synthesis of more selective biological-effect analogues is of paramount importance. In the title compound (Figure 1), the C3A—C7A shared bond of the bicyclic moiety presents a *trans* configuration. Besides, the 5-membered ring adopts an envelope conformation with puckering parameters *Q* = 0.462 (3) Å and φ = 136.5 (4)° and with the bridgehead C7A atom bearing the methyl substituent as the flap (Cremer & Pople, 1975) and the 6-membered ring presents a chair conformation with puckering parameters *Q* = 0.556 (3) Å, θ = 169.5 (3)° and φ = 133.4 (18)° (Cremer & Pople, 1975). The value for the dihedral angle between the idealized planes of the rings is 4.08 (17)°. All bond lengths and bond angles are normal comparable to those observed in similar crystal structures (Maehr & Uskokovic, 2004). In the crystal structure, the molecules are self-assembled *via* classical O—H···O hydrogen bonds to form a chain along [112], the resulting chains are connected by weak non-classical C—H···O hydrogen bonds to create a two-dimensional supramolecular structure (Table 1, Figure 2).

### Experimental

Over a stirring solution of inhoffen-lythgoe diol (2.1 g; 7.2 mmol) in CH_2_Cl_2_ (10 ml), PDC *S*-methyl-3-hydroxy-2-methylpropionate (5.4 g; 14.4 mmol) was added. The mixture was stirred at room temperature for 16 h then it was quenched with ethylic ether (20 ml) and stirred one more hour. The solid precipitated was filtered over celite, the organic layer was concentrated and the residue was purified by flash column chromatography on silica gel (10% ethyl acetate/hexane) to afford the title compound (1.8 g; 80%). The crystals were obtained by slow evaporation in a closed camera of a solution of the compound in a mixture of ethyl acetate/hexane (7:3).

### Refinement

All H-atoms were positioned and refined using a riding model with O–H = 0.82 Å and C–H = 0.98, 0.97, 0.96 and 0.93 Å for methine, methylelne, methyl and vinyl H-atoms, respectively. The H-atoms were allowed *U*_iso_ = 1.5*U*_eq_(O/C-methyl) or 1.2*U*_eq_(the rest of the C atoms). Due to insufficient anamolous dispersion effects, an absolute structure was not established in this analysis and 1457 Friedel pairs were not merged. However, the absolute configuration of the title compound was established according to the configuration of starting material.

### Crystal data

**Table:**

C_19_H_32_O_2_	*F*(000) = 648
*M**_r_* = 292.45	*D*_x_ = 1.035 Mg m^−^^3^
Monoclinic, *C*2	Mo *K*α radiation, λ = 0.71073 Å
Hall symbol: C 2y	Cell parameters from 1729 reflections
*a* = 20.057 (4) Å	θ = 2.2–23.0°
*b* = 7.3816 (15) Å	µ = 0.07 mm^−^^1^
*c* = 13.700 (3) Å	*T* = 293 K
β = 112.324 (4)°	Prism, colourless
*V* = 1876.3 (6) Å^3^	0.45 × 0.36 × 0.18 mm
*Z* = 4	

### Data collection

**Table:**

Bruker SMART 1000 CCD diffractometer	3254 independent reflections
Radiation source: fine-focus sealed tube	2389 reflections with *I* > 2σ(*I*)
Graphite monochromator	*R*_int_ = 0.018
φ and ω scans	θ_max_ = 25.0°, θ_min_ = 1.6°
Absorption correction: multi-scan (*SADABS*; Sheldrick, 1996)	*h* = −23→14
*T*_min_ = 0.602, *T*_max_ = 0.745	*k* = −8→8
4958 measured reflections	*l* = −13→16

### Refinement

**Table:**

Refinement on *F*^2^	Primary atom site location: structure-invariant direct methods
Least-squares matrix: full	Secondary atom site location: difference Fourier map
*R*[*F*^2^ > 2σ(*F*^2^)] = 0.048	Hydrogen site location: inferred from neighbouring sites
*wR*(*F*^2^) = 0.152	H-atom parameters constrained
*S* = 1.02	*w* = 1/[σ^2^(*F*_o_^2^) + (0.0934*P*)^2^ + 0.0697*P*] where *P* = (*F*_o_^2^ + 2*F*_c_^2^)/3
3254 reflections	(Δ/σ)_max_ < 0.001
196 parameters	Δρ_max_ = 0.14 e Å^−^^3^
1 restraint	Δρ_min_ = −0.11 e Å^−^^3^

### Special details

**Table:**

Geometry. All e.s.d.'s (except the e.s.d. in the dihedral angle between two l.s. planes) are estimated using the full covariance matrix. The cell e.s.d.'s are taken into account individually in the estimation of e.s.d.'s in distances, angles and torsion angles; correlations between e.s.d.'s in cell parameters are only used when they are defined by crystal symmetry. An approximate (isotropic) treatment of cell e.s.d.'s is used for estimating e.s.d.'s involving l.s. planes.
Refinement. Refinement of *F*^2^ against ALL reflections. The weighted *R*-factor *wR* and goodness of fit *S* are based on *F*^2^, conventional *R*-factors *R* are based on *F*, with *F* set to zero for negative *F*^2^. The threshold expression of *F*^2^ > σ(*F*^2^) is used only for calculating *R*-factors(gt) *etc*. and is not relevant to the choice of reflections for refinement. *R*-factors based on *F*^2^ are statistically about twice as large as those based on *F*, and *R*- factors based on ALL data will be even larger.

### Fractional atomic coordinates and isotropic or equivalent isotropic displacement parameters (Å^2^)

**Table:**

	*x*	*y*	*z*	*U*_iso_*/*U*_eq_	
C1	0.31315 (12)	−0.0108 (3)	0.83385 (16)	0.0467 (6)	
H1	0.3524	0.0312	0.8135	0.056*	
C2	0.32284 (17)	−0.2166 (4)	0.8512 (2)	0.0663 (8)	
H2A	0.3046	−0.2556	0.9039	0.080*	
H2B	0.3735	−0.2484	0.8752	0.080*	
C3	0.28017 (17)	−0.3084 (4)	0.7442 (2)	0.0737 (8)	
H3A	0.3107	−0.3896	0.7239	0.088*	
H3B	0.2394	−0.3759	0.7469	0.088*	
C3A	0.25554 (14)	−0.1495 (3)	0.66904 (18)	0.0554 (7)	
H3A1	0.2968	−0.1140	0.6516	0.066*	
C4	0.19282 (15)	−0.1736 (4)	0.5661 (2)	0.0619 (7)	
O4	0.16051 (12)	−0.3154 (3)	0.53886 (15)	0.0849 (7)	
C5	0.17509 (18)	−0.0054 (5)	0.4997 (2)	0.0807 (9)	
H5A	0.1294	−0.0224	0.4411	0.097*	
H5B	0.2118	0.0139	0.4708	0.097*	
C6	0.17031 (19)	0.1620 (5)	0.5617 (2)	0.0827 (10)	
H6A	0.1253	0.1584	0.5727	0.099*	
H6B	0.1695	0.2690	0.5202	0.099*	
C7	0.23275 (15)	0.1787 (4)	0.6691 (2)	0.0673 (8)	
H7A	0.2769	0.2040	0.6582	0.081*	
H7B	0.2236	0.2798	0.7075	0.081*	
C7A	0.24264 (12)	0.0069 (3)	0.73468 (16)	0.0472 (6)	
C8	0.17703 (13)	−0.0288 (5)	0.7620 (2)	0.0706 (8)	
H8A	0.1349	−0.0386	0.6982	0.106*	
H8B	0.1710	0.0694	0.8038	0.106*	
H8C	0.1838	−0.1397	0.8012	0.106*	
C1'	0.31086 (19)	0.2943 (4)	0.9234 (2)	0.0799 (10)	
H1'1	0.3417	0.3421	0.8905	0.120*	
H1'2	0.3227	0.3496	0.9914	0.120*	
H1'3	0.2615	0.3200	0.8801	0.120*	
C2'	0.32153 (14)	0.0889 (4)	0.93674 (19)	0.0552 (7)	
H2'	0.2857	0.0405	0.9624	0.066*	
C3'	0.39471 (14)	0.0496 (4)	1.01717 (17)	0.0554 (7)	
H3'	0.4331	0.0888	1.0004	0.067*	
C4'	0.41212 (13)	−0.0327 (4)	1.10809 (18)	0.0578 (7)	
H4'	0.3744	−0.0706	1.1268	0.069*	
C5'	0.48679 (13)	−0.0715 (4)	1.18480 (19)	0.0564 (7)	
H5'	0.5196	−0.0367	1.1503	0.068*	
C6'	0.4971 (2)	−0.2739 (5)	1.2070 (4)	0.1075 (14)	
H6'1	0.4895	−0.3375	1.1425	0.161*	
H6'2	0.4631	−0.3156	1.2359	0.161*	
H6'3	0.5452	−0.2962	1.2566	0.161*	
C7'	0.50827 (14)	0.0412 (5)	1.2868 (2)	0.0674 (8)	
O7'	0.58126 (10)	−0.0077 (3)	1.34707 (14)	0.0824 (7)	
H7'	0.5959	0.0494	1.4026	0.124*	
C8'	0.5057 (2)	0.2410 (5)	1.2604 (3)	0.1151 (17)	
H8'1	0.5409	0.2673	1.2307	0.173*	
H8'2	0.5159	0.3110	1.3235	0.173*	
H8'3	0.4585	0.2715	1.2104	0.173*	
C9'	0.4620 (2)	0.0049 (11)	1.3478 (3)	0.145 (2)	
H9'1	0.4765	0.0822	1.4086	0.218*	
H9'2	0.4672	−0.1194	1.3699	0.218*	
H9'3	0.4126	0.0287	1.3040	0.218*	

### Atomic displacement parameters (Å^2^)

**Table:**

	*U*^11^	*U*^22^	*U*^33^	*U*^12^	*U*^13^	*U*^23^
C1	0.0422 (12)	0.0519 (14)	0.0368 (10)	−0.0019 (12)	0.0048 (9)	−0.0014 (11)
C2	0.0731 (18)	0.0573 (17)	0.0500 (13)	0.0055 (14)	0.0027 (13)	−0.0010 (13)
C3	0.088 (2)	0.0542 (16)	0.0582 (15)	0.0030 (15)	0.0040 (14)	−0.0063 (14)
C3A	0.0536 (14)	0.0559 (16)	0.0444 (13)	−0.0055 (12)	0.0048 (11)	−0.0064 (11)
C4	0.0613 (16)	0.0701 (19)	0.0438 (13)	−0.0111 (15)	0.0079 (12)	−0.0117 (13)
O4	0.0868 (15)	0.0816 (15)	0.0583 (11)	−0.0232 (13)	−0.0039 (10)	−0.0162 (11)
C5	0.086 (2)	0.090 (2)	0.0425 (13)	−0.0127 (18)	−0.0011 (13)	0.0046 (16)
C6	0.093 (2)	0.077 (2)	0.0523 (16)	0.0033 (18)	−0.0011 (16)	0.0166 (15)
C7	0.0736 (19)	0.0615 (18)	0.0467 (14)	−0.0021 (16)	0.0002 (13)	0.0083 (13)
C7A	0.0444 (13)	0.0503 (14)	0.0385 (10)	−0.0030 (12)	0.0061 (9)	0.0007 (11)
C8	0.0474 (14)	0.101 (2)	0.0565 (14)	−0.0084 (15)	0.0117 (11)	−0.0040 (16)
C1'	0.095 (2)	0.072 (2)	0.0541 (15)	0.0132 (17)	0.0068 (15)	−0.0143 (14)
C2'	0.0507 (14)	0.0651 (18)	0.0419 (13)	−0.0001 (12)	0.0086 (11)	−0.0079 (11)
C3'	0.0508 (15)	0.0722 (19)	0.0359 (12)	−0.0071 (12)	0.0081 (11)	−0.0076 (11)
C4'	0.0484 (14)	0.0715 (19)	0.0470 (13)	−0.0112 (13)	0.0108 (11)	−0.0020 (13)
C5'	0.0483 (15)	0.0643 (18)	0.0478 (13)	−0.0007 (12)	0.0081 (11)	−0.0007 (12)
C6'	0.086 (3)	0.066 (2)	0.136 (3)	0.0032 (18)	0.003 (2)	0.007 (2)
C7'	0.0483 (15)	0.096 (3)	0.0433 (12)	0.0113 (14)	0.0012 (11)	−0.0076 (13)
O7'	0.0566 (12)	0.1072 (18)	0.0569 (10)	0.0184 (12)	−0.0083 (9)	−0.0136 (12)
C8'	0.097 (3)	0.082 (3)	0.112 (3)	0.018 (2)	−0.022 (2)	−0.034 (2)
C9'	0.094 (3)	0.287 (7)	0.0559 (17)	0.032 (4)	0.0299 (19)	0.005 (3)

### Geometric parameters (Å, º)

**Table:**

C1—C2	1.538 (4)	C8—H8C	0.9600
C1—C2'	1.541 (3)	C1'—C2'	1.533 (4)
C1—C7A	1.550 (3)	C1'—H1'1	0.9600
C1—H1	0.9800	C1'—H1'2	0.9600
C2—C3	1.545 (4)	C1'—H1'3	0.9600
C2—H2A	0.9700	C2'—C3'	1.491 (4)
C2—H2B	0.9700	C2'—H2'	0.9800
C3—C3A	1.515 (4)	C3'—C4'	1.308 (3)
C3—H3A	0.9700	C3'—H3'	0.9300
C3—H3B	0.9700	C4'—C5'	1.495 (3)
C3A—C4	1.501 (3)	C4'—H4'	0.9300
C3A—C7A	1.545 (3)	C5'—C6'	1.523 (5)
C3A—H3A1	0.9800	C5'—C7'	1.540 (4)
C4—O4	1.213 (3)	C5'—H5'	0.9800
C4—C5	1.500 (4)	C6'—H6'1	0.9600
C5—C6	1.524 (5)	C6'—H6'2	0.9600
C5—H5A	0.9700	C6'—H6'3	0.9600
C5—H5B	0.9700	C7'—O7'	1.427 (3)
C6—C7	1.532 (4)	C7'—C9'	1.490 (5)
C6—H6A	0.9700	C7'—C8'	1.515 (6)
C6—H6B	0.9700	O7'—H7'	0.8200
C7—C7A	1.524 (4)	C8'—H8'1	0.9600
C7—H7A	0.9700	C8'—H8'2	0.9600
C7—H7B	0.9700	C8'—H8'3	0.9600
C7A—C8	1.522 (3)	C9'—H9'1	0.9600
C8—H8A	0.9600	C9'—H9'2	0.9600
C8—H8B	0.9600	C9'—H9'3	0.9600
			
C2—C1—C2'	111.5 (2)	H8A—C8—H8B	109.5
C2—C1—C7A	103.77 (19)	C7A—C8—H8C	109.5
C2'—C1—C7A	120.50 (19)	H8A—C8—H8C	109.5
C2—C1—H1	106.8	H8B—C8—H8C	109.5
C2'—C1—H1	106.8	C2'—C1'—H1'1	109.5
C7A—C1—H1	106.8	C2'—C1'—H1'2	109.5
C1—C2—C3	107.2 (2)	H1'1—C1'—H1'2	109.5
C1—C2—H2A	110.3	C2'—C1'—H1'3	109.5
C3—C2—H2A	110.3	H1'1—C1'—H1'3	109.5
C1—C2—H2B	110.3	H1'2—C1'—H1'3	109.5
C3—C2—H2B	110.3	C3'—C2'—C1'	109.5 (2)
H2A—C2—H2B	108.5	C3'—C2'—C1	108.5 (2)
C3A—C3—C2	103.0 (2)	C1'—C2'—C1	113.7 (2)
C3A—C3—H3A	111.2	C3'—C2'—H2'	108.3
C2—C3—H3A	111.2	C1'—C2'—H2'	108.3
C3A—C3—H3B	111.2	C1—C2'—H2'	108.3
C2—C3—H3B	111.2	C4'—C3'—C2'	128.7 (3)
H3A—C3—H3B	109.1	C4'—C3'—H3'	115.6
C4—C3A—C3	119.2 (2)	C2'—C3'—H3'	115.6
C4—C3A—C7A	111.7 (2)	C3'—C4'—C5'	126.3 (2)
C3—C3A—C7A	105.5 (2)	C3'—C4'—H4'	116.8
C4—C3A—H3A1	106.6	C5'—C4'—H4'	116.8
C3—C3A—H3A1	106.6	C4'—C5'—C6'	110.6 (2)
C7A—C3A—H3A1	106.6	C4'—C5'—C7'	113.2 (2)
O4—C4—C5	123.5 (2)	C6'—C5'—C7'	112.2 (3)
O4—C4—C3A	123.4 (3)	C4'—C5'—H5'	106.8
C5—C4—C3A	113.1 (2)	C6'—C5'—H5'	106.8
C4—C5—C6	112.5 (2)	C7'—C5'—H5'	106.8
C4—C5—H5A	109.1	C5'—C6'—H6'1	109.5
C6—C5—H5A	109.1	C5'—C6'—H6'2	109.5
C4—C5—H5B	109.1	H6'1—C6'—H6'2	109.5
C6—C5—H5B	109.1	C5'—C6'—H6'3	109.5
H5A—C5—H5B	107.8	H6'1—C6'—H6'3	109.5
C5—C6—C7	113.5 (3)	H6'2—C6'—H6'3	109.5
C5—C6—H6A	108.9	O7'—C7'—C9'	110.5 (3)
C7—C6—H6A	108.9	O7'—C7'—C8'	108.6 (3)
C5—C6—H6B	108.9	C9'—C7'—C8'	109.6 (4)
C7—C6—H6B	108.9	O7'—C7'—C5'	105.1 (2)
H6A—C6—H6B	107.7	C9'—C7'—C5'	113.1 (3)
C7A—C7—C6	112.0 (2)	C8'—C7'—C5'	109.7 (3)
C7A—C7—H7A	109.2	C7'—O7'—H7'	109.5
C6—C7—H7A	109.2	C7'—C8'—H8'1	109.5
C7A—C7—H7B	109.2	C7'—C8'—H8'2	109.5
C6—C7—H7B	109.2	H8'1—C8'—H8'2	109.5
H7A—C7—H7B	107.9	C7'—C8'—H8'3	109.5
C8—C7A—C7	111.0 (2)	H8'1—C8'—H8'3	109.5
C8—C7A—C3A	111.4 (2)	H8'2—C8'—H8'3	109.5
C7—C7A—C3A	106.98 (19)	C7'—C9'—H9'1	109.5
C8—C7A—C1	110.87 (18)	C7'—C9'—H9'2	109.5
C7—C7A—C1	117.3 (2)	H9'1—C9'—H9'2	109.5
C3A—C7A—C1	98.54 (18)	C7'—C9'—H9'3	109.5
C7A—C8—H8A	109.5	H9'1—C9'—H9'3	109.5
C7A—C8—H8B	109.5	H9'2—C9'—H9'3	109.5
			
C2'—C1—C2—C3	153.7 (2)	C2—C1—C7A—C8	75.8 (3)
C7A—C1—C2—C3	22.6 (3)	C2'—C1—C7A—C8	−49.9 (3)
C1—C2—C3—C3A	6.2 (3)	C2—C1—C7A—C7	−155.3 (2)
C2—C3—C3A—C4	−159.6 (3)	C2'—C1—C7A—C7	79.0 (3)
C2—C3—C3A—C7A	−33.2 (3)	C2—C1—C7A—C3A	−41.2 (2)
C3—C3A—C4—O4	−0.6 (5)	C2'—C1—C7A—C3A	−166.8 (2)
C7A—C3A—C4—O4	−124.0 (3)	C2—C1—C2'—C3'	59.0 (3)
C3—C3A—C4—C5	−179.5 (3)	C7A—C1—C2'—C3'	−179.0 (2)
C7A—C3A—C4—C5	57.1 (3)	C2—C1—C2'—C1'	−178.8 (3)
O4—C4—C5—C6	132.6 (3)	C7A—C1—C2'—C1'	−56.9 (3)
C3A—C4—C5—C6	−48.5 (4)	C1'—C2'—C3'—C4'	117.4 (3)
C4—C5—C6—C7	46.1 (4)	C1—C2'—C3'—C4'	−118.0 (3)
C5—C6—C7—C7A	−52.5 (4)	C2'—C3'—C4'—C5'	178.7 (3)
C6—C7—C7A—C8	−63.9 (3)	C3'—C4'—C5'—C6'	−122.4 (4)
C6—C7—C7A—C3A	57.8 (3)	C3'—C4'—C5'—C7'	110.6 (3)
C6—C7—C7A—C1	167.2 (2)	C4'—C5'—C7'—O7'	−177.4 (2)
C4—C3A—C7A—C8	61.0 (3)	C6'—C5'—C7'—O7'	56.5 (3)
C3—C3A—C7A—C8	−69.8 (3)	C4'—C5'—C7'—C9'	62.0 (4)
C4—C3A—C7A—C7	−60.5 (3)	C6'—C5'—C7'—C9'	−64.2 (4)
C3—C3A—C7A—C7	168.7 (2)	C4'—C5'—C7'—C8'	−60.8 (3)
C4—C3A—C7A—C1	177.5 (2)	C6'—C5'—C7'—C8'	173.1 (3)
C3—C3A—C7A—C1	46.7 (2)		

### Hydrogen-bond geometry (Å, º)

**Table:**

*D*—H···*A*	*D*—H	H···*A*	*D*···*A*	*D*—H···*A*
O7′—H7′···O4^i^	0.82	2.08	2.876 (3)	164
C3*A*—H3*A*1···O7′^ii^	0.98	2.56	3.523 (3)	166

Symmetry codes: (i) *x*+1/2, *y*+1/2, *z*+1; (ii) −*x*+1, *y*, −*z*+2.
